# Scoring functions for drug-effect similarity

**DOI:** 10.1093/bib/bbaa072

**Published:** 2020-06-02

**Authors:** Stephan Struckmann, Mathias Ernst, Sarah Fischer, Nancy Mah, Georg Fuellen, Steffen Möller

**Affiliations:** 1 IBIMA, Rostock University Medical Center, Rostock, 18041, Germany; 2 SHIP-KEF, Institute for Community Medicine, University Medicine of Greifswald, Walther-Rathenau-Straβe 48, 17475 Greifswald, Germany; 3 Friedrich-Alexander-University Erlangen-Nuremberg, 91058 Erlangen, Germany; 4 BCRT - Berlin Institute of Health Center for Regenerative Therapies, Charité - University Medicine Berlin, 13353, Germany

**Keywords:** drug repurposing, drug repositioning, transcriptomics, evaluation

## Abstract

**Motivation:**

The difficulty to find new drugs and bring them to the market has led to an increased interest to find new applications for known compounds. Biological samples from many disease contexts have been extensively profiled by transcriptomics, and, intuitively, this motivates to search for compounds with a reversing effect on the expression of characteristic disease genes. However, disease effects may be cell line-specific and also depend on other factors, such as genetics and environment. Transcription profile changes between healthy and diseased cells relate in complex ways to profile changes gathered from cell lines upon stimulation with a drug. Despite these differences, we expect that there will be some similarity in the gene regulatory networks at play in both situations. The challenge is to match transcriptomes for both diseases and drugs alike, even though the exact molecular pathology/pharmacogenomics may not be known.

**Results:**

We substitute the challenge to match a drug effect to a disease effect with the challenge to match a drug effect to the effect of the same drug at another concentration or in another cell line. This is welldefined, reproducible *in vitro* and *in silico* and extendable with external data. Based on the Connectivity Map (CMap) dataset, we combined 26 different similarity scores with six different heuristics to reduce the number of genes in the model. Such gene filters may also utilize external knowledge e.g. from biological networks. We found that no similarity score always outperforms all others for all drugs, but the Pearson correlation finds the same drug with the highest reliability. Results are improved by filtering for highly expressed genes and to a lesser degree for genes with large fold changes. Also a network-based reduction of contributing transcripts was beneficial, here implemented by the FocusHeuristics. We found no drop in prediction accuracy when reducing the whole transcriptome to the set of 1000 landmark genes of the CMap’s successor project Library of Integrated Network-based Cellular Signatures. All source code to re-analyze and extend the CMap data, the source code of heuristics, filters and their evaluation are available to propel the development of new methods for drug repurposing.

**Availability:**

https://bitbucket.org/ibima/moldrugeffectsdb

**Contact:**

steffen.moeller@uni-rostock.de

**Supplementary information:**

Supplementary data are available at *Briefings in Bioinformatics* online.

## 1 Introduction

The introduction of high-throughput transcriptome profiling technology (e.g. DNA microarrays and RNA-sequencing) has enabled scientists to gather insights on how sets of genes are dysregulated in human diseases. However, these observations alone cannot be used to deduce how to revert an aberrant disease transcriptome signature towards a healthy state, i.e. these do not suggest a treatment by themselves. Two major issues arise: (i) how to assess a dataset’s suitability to measure the effect of molecular treatments on the transcriptome; (ii) how should molecular effects of treatments be matched with the molecular effects of diseases in a suitable way?

Over the past two decades, advances in automation and miniaturization have led to the development of Connectivity Map [29] (CMap), an invaluable data collection of microarray expression profiles of five cancer cell lines in response to 1300 small bioactive molecules, including Food and Drug Administration–approved drugs, see [25]. CMap was the basis for the Library of Integrated Network-based Cellular Signatures (LINCS) L1000 project [12, 43], which greatly expanded the number of tested perturbagens (including small molecules, shRNA and biologics) to 25200 in nine cancer or immortalized cell lines. Public gene expression databases, notably ArrayExpress [3] and Gene Expression Omnibus [5], offer additional insights on drug effects in more than 6500 experiments. Specific toxicogenomic resources, such as DrugMatrix [18], the Comparative Toxicogenomics Database [10] and Open TG-GATES [22], have been established with the aim to examine the effects of drug-induced toxicity. Some years ago, this journal provided an excellent overview on the general concepts for gene-expression-based drug repurposing [32].

With better insights on the pharmacogenomics of drugs, new ideas have emerged for repurposing compounds that are already on the market, i.e. for those that have already undergone the expensive process of clinical testing. An algorithm to filter drugs for the changes to gene expression they induce was already proposed with the first CMap paper [29] in 2006. The field has matured and secondary databases have been developed that curate clinical trial data for successful and unsuccessful repurposing efforts [7], from which methods for drug effect predictors can learn, or that curate data from a variety of other clinical trial databases [20]. Phenotypes that are clinically diverse to describe, such as aging [11], are now subjected to repurposing. For the many patients suffering from rare diseases, to repurpose existing drugs may be considered the shortest path to improve their condition [42]. The comparison of drug effects with molecular characterizations of disease enables individualized medicine in a natural way.

The more we know how to best match the changed expression of genes under a disease with changes induced by a drug, the more useful the CMap and its successor LINCS will become. The description of the effects of a compound on the transcriptome could then nicely extend the description of otherwise yet uncharacterized compounds in public databases such as ChEMBL [19] and PubChem [26]. In addition to the steadily falling costs of generating such data *de novo*, a considerable wealth of data is stored in expression databases such as ArrayExpress and Gene Expression Omnibus, but these are not yet readily available for transcriptome-based similarity searches. Efforts such as MEM [1] integrate these data—but for a different kind of cross-experiment comparison on single gene expression levels. Once drug effect data are better understood, successors to the routines presented in this paper are likely to be integrated towards sophisticated web services to guide repurposing research.

With only the effect on the transcriptome known, but not the compounds’ protein targets, virtual screening techniques based on chemical / structural data are complementary tools for repurposing. For example, the chemical properties of compounds can be used to derive pharmacophores, which are suggestive for additional compounds to investigate. These technologies and data are increasingly available to be integrated into accompanying workflows [4, 35, 39].

While these datasets represent a wealth of information, the application of finding drugs with potentially beneficial effects to a particular disease presents a challenge. Ideally, we would like to identify drugs that suppress the genes contributing to the disease and increase the expression of genes that alleviate the disease. However, transcriptomic snapshots cannot distinguish cause from effect—for example, which genes are causative for a disease and which are in physiological feedback loops, activated in response to disease processes? A good overall matching/reversal should necessarily indicate the identification of a good drug candidate. As an alternative, one could integrate data from complementary sources like established molecular networks and statistical genetics to direct the selection of disease-relevant genes.

Of the many genes changed in their expression by a disease, not all are of the same importance for a phenotypic effect. Also, many strongly co-regulated genes do not offer additional insights but give a bias over a single representant of such a cluster. We therefore propose to use a reduced set of genes for the comparison, which could increase the robustness of any method used to find similarities thereafter.

In the present study, the CMap raw data were reanalyzed and presented in a way that preserves the redundancy in drug effects across the different microarray platforms, cell lines and drug concentrations. Our hypothesis is that any computational approach that aspires to match drugs to diseases by comparing transcriptional profiles should also manage to match the drug to the expression profile of the same drug, just with that drug measured under different conditions, but with the same genes that are primarily affected.

We implemented routines to filter for genes, i.e. for their high expression in a cell line, for their high fold changes (FCs) with respect to baseline expression, high $Z$ score or low $P$ value. Additionally, we performed a network-based selection of genes with our FocusHeuristic [16]. This array of options for gene filtering was systematically evaluated for their effects on the similarity scores to identify the same drug in another concentration or tissue or platform only based on changes in gene expression levels.

This work supports the community to quickly assess the performance of heuristics to match expression profiles that share the same molecular trigger. We believe that this is an easier problem than matching a drug effect to disease expression data due to the confounding effects of tissue heterogeneity and polygenicity. As we show, it is already difficult enough to clearly separate similarity scoring functions and gene filters by their performance.

## 2 Approach

**Figure 1:**
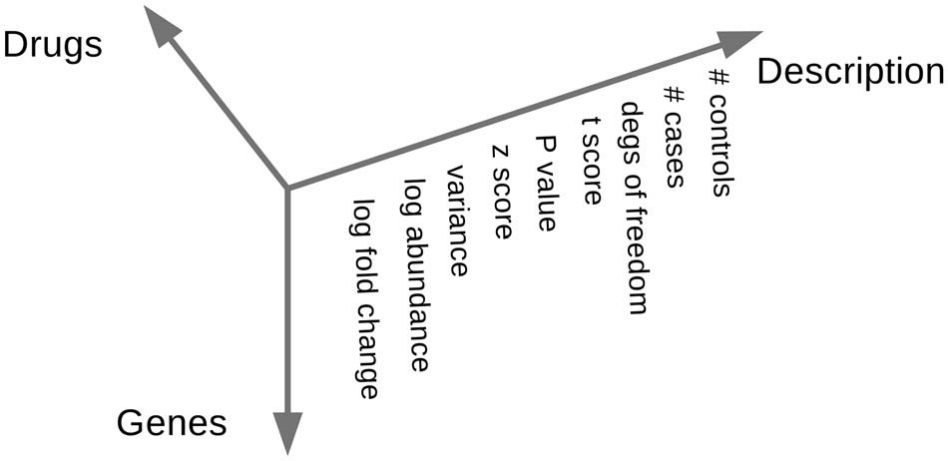
Data prepared for each technological platform of CMap. The CMap data are presented as a list of three matrices, each representing a technological platform (U133AAofAv2, HG-U133A, HT_HG-U133A). The matrices are three-dimensional. All show the same subset of Entrez [46] genes as rows. Every drug at each concentration for each cell line represents a column. A third dimension offers log fold changes, absolute expression levels (log abundance) and information on reliability gathered from an analysis of the technical replicates: their variance, $Z$ score, the $P$ value from a $t$ test against the untreated cell culture, the $t$ score of that test, degrees of freedom and the number of cases (replicates of treatment with the drug) and controls available for that drug at that concentration.

For this project, the raw Affymetrix data of the CMap were formatted into the data structure presented in Figure 1. This CMap data structure offers 3675 instances of drugs tested on a particular combination of cell line, concentration and platform.

For this work, we assess several scoring functions of drug similarity by their success rate to find the same drug in another instance, i.e. despite the drug’s concentration, cell line or platform being different. This is possible for a subset of 1549 instances of the total of 3675 that refer to a drug that was tested in multiple conditions. The difficulty to identify the same drug we expect to depend on the number of genes changed by the drug and their specificity for that drug.

All instances are treated in the same way for this analysis, disregarding the number of instances covering the same drug. We review only basic scoring functions that work context free, i.e. that do not benefit by learning from molecular effects of other instances in CMap.

**Figure 2:**
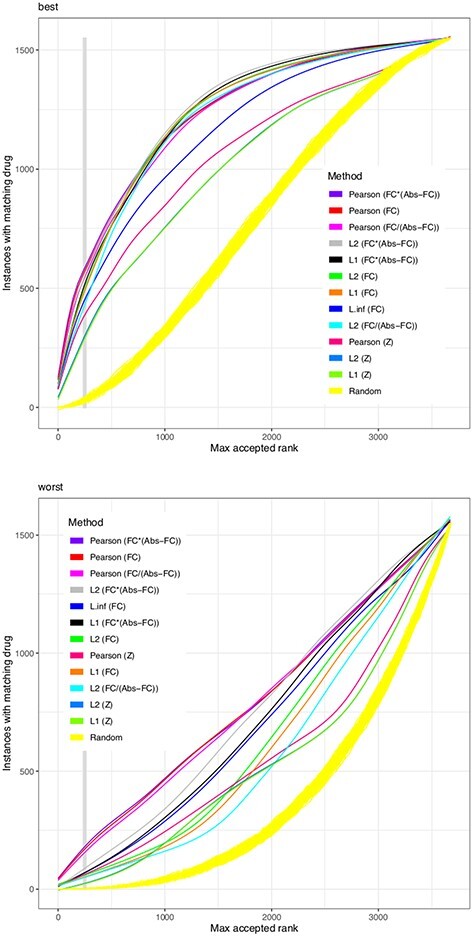
Similarity scores based on the FC. The figure indicates the performance of several similarity scores, each represented in a different color. For each drug-platform-cell line-concentration instance in CMap, based only on pairwise comparisons of the change in gene expression, a similarity score yields a ranking for all other instances. The X axis represents a threshold, i.e. a maximal rank up to which an instance shall be accepted to contribute to the evaluation. In the upper plot, the Y axis indicates the number of all CMap instances that have at least one occurrence where the respective same drug was observed below (i.e., ranking better than) that maximal rank $x$. In the lower plot, the Y axis counts the number of instances for which *all* occurrences of the same drug are below that maximal rank. The yellow streak results from 100 invocations of a scoring function returning random numbers. While all scores perform better than random, the Pearson correlation is the most successful. The legend is sorted according to the performance of each scoring function, from best to worst performance, at a maximal rank of 250 (indicated by the vertical gray bar).

**Figure 3:**
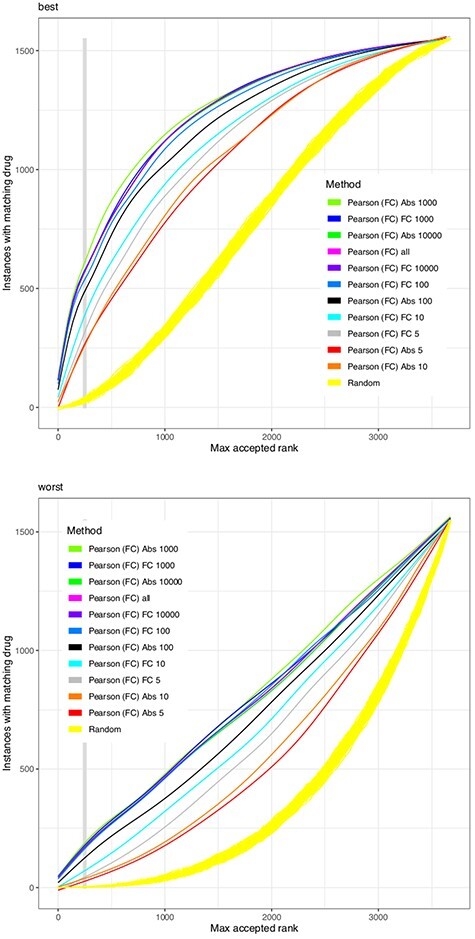
Abundance and change as gene filters. Analogously to Figure 2, the Pearson correlation as a similarity function is run on different subsets of genes. Genes are filtered for their maximal abundance, selecting the 5, 10, 100 and 10 000 most expressed (‘Abs $n$’) or most changing genes (‘FC $n$’) in comparison with the performance of the complete set (‘all’). With either filter, at around 1000 genes is a maximum of the performance.

**Figure 4:**
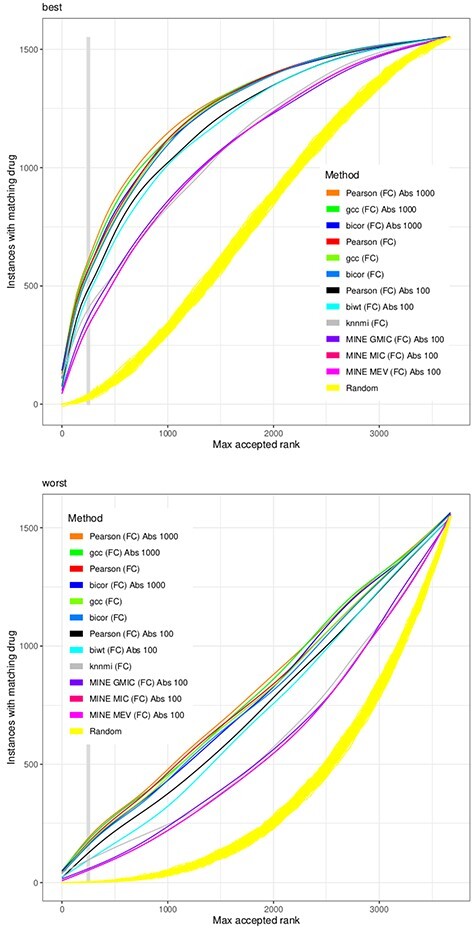
Similarity functions from software packages for calculating gene expression correlation networks. Pearson is provided for reference, see also the preceding figure. MINE-GMIC, MINE-MIC and MINE-MEV represent the many mutual information routines by the minerva package. biwt and the mine routines are computationally too expensive to be evaluated on the full set of genes.

**Figure 5:**
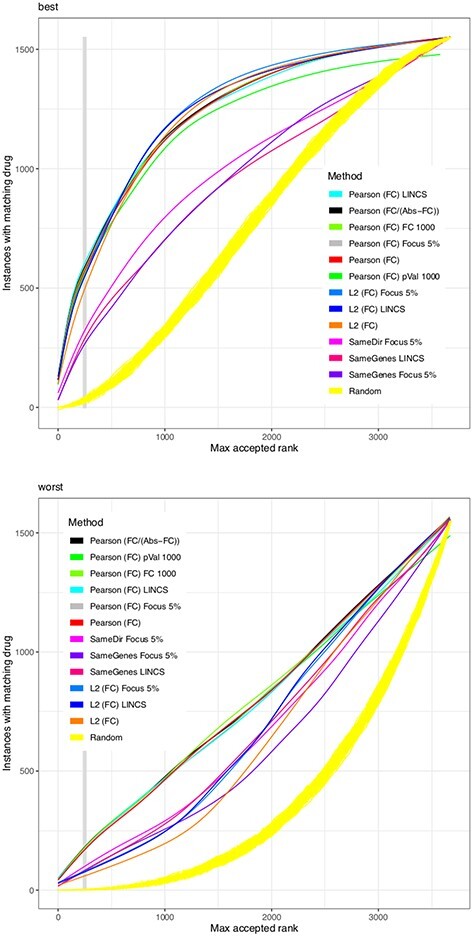
External data contributing to selection of 1000 genes. Close to 1000 genes were selected by the CMap successor, LINCS, to represent drug responses. The FocusHeuristics (``Focus'') reduces gene interaction networks on the basis of gene expression changes, here run with parameters to also yield a reduction to 1000 genes. These filters are shown in the context of the Pearson and $L_2$ similarity scores. The ‘same dir’ scoring function counts the number of genes changing to the same direction with a log ratio > 0.5 in both instances.

All scoring functions are applied on every instance in the above described subset of 1549 CMap instances to compare these with all 3675 instances available. By sorting the similarity scores for each instance, we assign a first (‘best’) position at which the same drug (at different concentration/cell line/platform) is reoccurring and a last (worst) position. Figures 2 to 5 show, for different scoring functions, on the Y axis the number of instances for which the same drug is among the maximum accepted ranks set by the threshold on the X axis.

## 3 Methods

All data processing was performed with the R statistics suite [37]. CMap raw data from Affymetrix.CEL files and their annotations (from https://portals.broadinstitute.org/cmap) were converted to a three-dimensional data frame (Figure 1). The microarray expression data were normalized using RMA and adjusted for batch effects using the ComBat function of the sva R package [31]. Supported by the annotation file provided by CMap, the drug names were mapped to PubChem [26] identifiers. Gene names were mapped using BioConductor’s org.Hs.eg.db [8] database and completed with BioMart [13, 14, 27] to minimize the number of unmapped transcripts. Plots were implemented with help of the ggplot2 R package [47].

Similarity scoring functions were implemented for the $L_1$ ‘Manhattan’ norm ($|x-y|$), $L_2$ ‘Euclidian’ norm ($\sqrt{(x-y)^2}$) and the $L_{\infty }$ ‘maximum’ norm ($max(x,y)$). Kolmogorov–Smirnov test and Pearson and Spearman correlation were carried out using the R statistics suite [37]. Jaccard scores were implemented to work on either absolute or relative changes in gene expression, with an optional additional constraint on genes to change in the ‘same direction’ (both up- or both down-regulated). FCs in gene expression were defined to be the log2 ratio between treated compared to untreated (control) conditions. As a control we analogously implemented a Jaccard score with the constraint on genes to change to opposite directions. From the rsgcc R package the Gini method [33] was tested (gcc). Biweight midcorrelation was implemented by the WGCNA [30] and the biwt [21] packages. Mutual information measures were contributed by the R packages parmigene [40] (knnmi) and minerva [2]. See Supplement Table 1 for an overview.

Figures 2 to 5 use the abbreviations ‘FC’ in parentheses to indicate that the scoring function was applied on the gene expression log ratio directly. ‘FC*(Abs-FC)’ has the log ratio multiplied with the expression level in the control samples. ‘FC/(Abs-FC)’ puts the change in relation to the expression level.

For gene filtering we applied the FocusHeuristic [16] as a reference for network-based approaches (employing the human STRING 10 network [44] with combined score above 0.9 as extracted using the STRINGdb R package [17]) and implemented filters for the highest expressed or most changing genes. The 1000 landmark genes of the CMap project’s successor LINCS were retrieved from the annotation of the NCBI GEO dataset GSE92742 ’gene info’.

## 4 Results and Discussion

This presentation of the CMap data offers multiple (see Figure 1) numerical values per gene to describe the change in expression upon exposure of a drug and a statistical description of the confidence in the change to be real. The use of these values is 2-fold. First, these values can be used to filter for genes that are most informative for a comparison, i.e. to direct the search to the genes that are most characteristic for each drug. Second, these values are also the input for similarity scoring functions. While the (logarithmic) FC by itself is a prominent input for similarity scoring, its combination with the absolute expression level of a gene or comparing with the predictivity of a random variation of expression levels allows for additional insights. This study shows in supplementary Tables 2 and 3 that for integrating absolute expression data it makes no practical difference whether the absolute expression levels of untreated cells (methods have suffix ‘corrected’ in their name) or treated cells are used.

The performance of a scoring function is evaluated by the number of instances that have a matching drug (y axis) among the most similar expression profiles when testing the first $x$ ranks, as set by the threshold of maximum accepted ranks. The Pearson correlation performs best (Figure 2) followed by the $L_2$ norm. Differences become more apparent when inspecting the ‘worst-case’ performance, i.e. the lowest ranking position at which the same drug is ranked. Moreover, the performance of $z$ scores was not found to be superior over using FCs directly, suggesting that a low variance of gene expression in response to a drug is not indicative of a gene’s contribution. Also, when applying similarity scores on FCs, filtering for genes with absolute expression performs better than for genes on lowest $P$ values, highest $z$ scores or the highest FCs themselves. The Kolmogorov–Smirnov test compares the distribution of changes alone, much like a comparison by Q-Q plots, and does not shine in this evaluation (‘rel.ks’ in supplementary tables).

We find that $L_1$ and $L_2$ benefit from being run on the differences of *non-logarithmic* expression levels (scoring functions with ‘raw’ in their name, compare Supplementary Tables 1 and 2). This may be explained by the larger numeric difference of the most-changing transcripts versus the rest—as if this was an intrinsic filter for significance. (The logarithmic representation is needed to achieve a symmetry for up- and down-regulation.)

Predictions improve with more genes contributing to the comparison of drug effects, but fewer than 100 genes already yield near-optimal results when using only the most highly expressed genes (Figure 3). The methods SameGenes and SameDir, counting the number of genes jointly affected/changing to the same direction, were found to be non-competitive. The Jaccard implementation normalizes above-mentioned counts by the overall number of genes changed in the dataset. It was found to be the most robust scoring function, when sorting for the last rank of the same drug to be found. (With filters on genes applied first, this leads to a division by zero error when thresholds to recognize a differential expression are too strict and no genes are found to be differently expressed.)

The methods used for generating gene expression correlation networks are not performing better than the Pearson correlation (Figure 4). The scorings of bicor, gcc and Pearson are similar. The performance of mutual information scoring functions of the minerva package is inferior, despite the considerable additional computational effort these require. The methods did not benefit from being applied on the $z$ score instead of the log ratio and also the constraint on ‘the genes that are also used by LINCS’ does not improve their performance (see tables in supplement).

Similarity scoring functions tested in this analysis can be applied to describe the similarity of any pair of expression profiles, for any subset of genes. Their selection we consider to be reasonably complete with respect to routines that are easily implemented or directly offered by R and are context free. The Pearson correlation returned the same ranks as our implementation of the cosine measure [15].

While every scoring function we tested performed better than random, the different approaches did not correlate with each other but were most successful on different drugs (Supplementary Figure 6). The scoring functions were applied to the CMap data that were directly derived from the ‘.CEL’ files after correcting for batch effects (Figure 1). Most of these functions however are completely agnostic about the nature of the data presented and as such would also be applicable on transformations of that data. For instance, after mapping the CMap expression data to a gene–protein interaction network, the transformed data could represent scores for connections between genes instead of scores for the genes themselves. Or, a principal component analysis could be performed and the similarity scores could then be calculated based on the loadings of the instances.

Not all genes need to be considered as an input for the similarity score. Figure 5 indicates that filtering for the most expressed or most changed genes improves the performance. The integration of network data by the FocusHeuristics brings good improvements, e.g. to the $L_2$ scoring function. But while improving the results of the Pearson correlation, there is no advantage over using simpler gene filters. When only considering those genes that act as representations of correlating gene sets, as performed by the LINCS project, this is also beneficial. It should be noted that the gene selection by LINCS is static, i.e. it is not adjusted to the data observed in the individual drug experiment.

Historically, the roots for matching compounds with a desired effect from a large database with known targets in clinical applications lie in structural biochemistry. With the increased availability of transcriptomic profiles, the repurposing of drugs is increasingly based on comparing effects on gene regulation. As a consequence, this alleviates the need to single out explicit drug targets—the comparison is performed on the cell line’s systemic effect. The CMap [29] is still the most popular systematic data collection of drug effects on the transcriptome. Its successor project LINCS [12, 43] has dramatically reduced the number of transcripts from a whole transcriptome approach down to 1000 genes. Here we have shown (Figure 5) that the reduction to this LINCS set of genes is not expected to reduce the performance of drug-effect predictions. All methods tested perform better on the 1000 most expressed genes than on the most expressed 100 genes. But their performance is slightly reduced when all genes are input to the similarity scoring function.

We are not aware of scores that work on gene regulatory features. These could be derived from static data, e.g. as presented with g:Profiler [38] or enrichr [28], or from deep learning efforts like ExPecto [51]. The integration of the right similarity functions, filters and features for matching expression profiles and diseases may be a challenge for deep learning itself.

Our effort on the CMap whole transcriptome data could be repeated for the 1000-gene datasets from the much more diverse LINCS project. The scoring functions perform better on its reduced gene set, but the challenge to identify the same drug in a set of drug effects is still difficult enough. Even with a completely independent implementation on other datasets, the Pearson scoring was found robust in its performance when gene filters were applied or the absolute expression levels were integrated. The Pearson correlation may hence serve as a reference to determine the gain by new approaches to map drugs to changes in expression profiles.

The wet-lab work required for proving or disproving a predicted drug effect is considerable. When only few wet-lab experiments can be afforded to prove a finding, our analyses indicate that it is unlikely to match a drug’s effect across cell lines, concentrations and technical platforms: from the upper diagram of Figures 2 to 5 we can derive that only 20% of instances can find a match within the first 100 ranks. This success rate matches findings by Pabon and coworkers on the LINCS dataset [36].

Therefore, these data suggest that additional information or better predictors are required to be able to make use of CMap-based repurposing in the wet-lab. The scoring functions presented in this study already contribute to the workflows of repurposing tools such as Cogena [24], Dr Insight [9] and NFFinder [41], embedded in various kinds of pre- and/or post-processing, such as clustering or gene set enrichment analyses. One may of course ask what part of the workflow is contributing how much to the quality of the findings, and why? For instance, a motivation for the development of Dr Insight was to dispense with any need to predetermine the length of a gene query signature (that is, the size of the subset of characteristic genes). In our study we show in Figure 5 that indeed the quality of predictions increases with more input genes. Most likely, also other parameters of a complex method such as Dr Insight could be evaluated and potentially be improved, based on a systematic evaluation in a setup as demonstrated in the current study.

Our approach to assess scoring functions can also be used for evaluating machine learning (ML)-based methods for drug repositioning. We require no external (literature or database) metadata about the similarity of diseases or the shared structural properties of the compounds, as seen in some recently published evaluations such as ‘DeepSide’ [45] or ‘DeepDR’ [50]. This way, the challenge is to find the same drug only by its transcriptional fingerprint—across concentrations, technical platforms and tissues. Applied to evaluating the quality of models based on ML, the rank(s) at which the transcriptomic effects of the same drugs are evaluated can be used as a semi-quantitative indicator of prediction quality.

ML approaches likely can be adapted to outperform the context-free scoring functions addressed in this review [49]. We used an objective evaluation based on the multiplicity of same-drug instances of CMap to investigate the effect of settings such as the contribution of network data and the reduction of the number of genes, on performance of predicting drug similarity. Analogously, such an objective assessment will also be possible and useful for analyzing the performance of emerging ML methods, and how these are affected by such settings.

## 5 Conclusion

In summary, our work provided some interesting accomplishments and insights such as the following. The re-analysis of the CMap.CEL files provides a resource to assess the performance of methods to match drug effects with molecular characteristics of diseases. The performance of all tested basic similarity scores is better than random. The best performing score is the Pearson correlation applied on FCs combined with a filter for the 1000 most changing genes. The performance of all tested similarity scores is improved by multiplying the FC with the expression level in the control experiments. The reduced set of genes of CMap successor LINCS has a positive effect on all tested scoring functions’ performance.

The mapping of drugs to diseases by their effects on gene expression levels is related to the mapping of diseases to each other, e.g. for co-morbidities or shared clinical symptoms, suggesting that one may refer to external data to filter for the presumably most-relevant genes [6, 34]. One may for instance refer to genetic insights such as from genome-wide association studies. An inspiring contribution is the tissue-effect database TSEA [23], facilitating insights from tissue-specific drug effect studies pertaining to diseases. It will be very interesting to watch the development of this integrative field.

In the future, the mere mapping of drugs with diseases will be superseded by a need to interpret molecular drug effects. From the perspective of individualized medicine, there will be a high demand to customize drug combinations and/or optimized drug treatments, based on the patient’s drug response profile and genetic constitution. The information to enable the meta-analysis of diverse datasets (e.g. transcriptomic, chemoinformatic, genomic) will necessitate FAIR data practices, which facilitate the sharing and usage of machine-readable datasets [48]. This work exemplifies how an increased FAIRness of CMap may contribute to better data practices. Coupled with models for cellular differentiation and/or the ongoing developments of personalized *in vitro* disease models, the concepts outlined in the present study work towards an integrated therapeutic approach.

##

**Key Points**

- Reanalysis of Connectivity Map (CMap) whole-genome transcriptome data to elucidate effects of the same drug in different cell lines, concentrations or when measured on different platforms.
- Use of the CMap dataset to review a series of similarity scores and gene filters on their ability to find the same drug only by comparing the effects on the transcriptomes.
- The reduction from whole-genome to a subset of 1000 genes in the CMap successor Library of Integrated Network-based Cellular Signatures had no negative effect on the performance of scoring functions.
- Setup provides an infrastructure to objectively evaluate future means for drug repurposing.

## Supplementary Material

AUTHORS_BIOGRAPHY_agaa047

## Notes

**Stephan Struckmann, Mathias Ernst, Sarah Fischer and Steffen Möller** are researchers at the Institute for Biostatistics and Informatics in Medicine and Aging Research (IBIMA) at the Rostock University Medical Center under the direction of Georg Fuellen. The research group focuses on the bioinformatic analysis of aging processes, based on laboratory and expression data of humans, mice and nematodes.

**Nancy Mah** is a researcher at the Berlin Institute of Health Center for Regenerative Therapies at the Charité Universitätsmedizin.
