# Supplementary material for: Scoring functions for drug-effect similarity
Source: Brief Bioinform. 2020 Jun 2;22(3):bbaa072. doi: 10.1093/bib/bbaa072 (PMC8138836; doi:10.1093/bib/bbaa072)
Supplement: AUTHORS_BIOGRAPHY_agaa047 [file authors_biography_agaa047.docx]

**AUTHORS BIOGRAPHY**

Stephan Struckmann and Steffen Möller are post-doctoral researchers in
Bioinformatics in the IBIMA institute of the Rostock University Medical
Center.
Headed by Georg Fuellen, as a computational group, the IBIMA
teams up with pre-clinical researchers of different disciplines for the
joint
investigation of common molecular motifs that are associated with aging
and the development of diseases. A special interest is on comparing drug
effects with disease expression data and statistical genetics.
